# Moving the Cellular Peptidome by Transporters

**DOI:** 10.3389/fcell.2018.00043

**Published:** 2018-04-30

**Authors:** Rupert Abele, Robert Tampé

**Affiliations:** ^1^Institute of Biochemistry, Biocenter, Goethe University Frankfurt, Frankfurt, Germany; ^2^Cluster of Excellence – Macromolecular Complexes, Goethe University Frankfurt, Frankfurt, Germany

**Keywords:** ABC transporter, antigen processing, antigen presentation, membrane proteins, viral immune escape, lysosome, endoplasmic reticulum

## Abstract

Living matter is defined by metastability, implying a tightly balanced synthesis and turnover of cellular components. The first step of eukaryotic protein degradation via the ubiquitin-proteasome system (UPS) leads to peptides, which are subsequently degraded to single amino acids by an armada of proteases. A small fraction of peptides, however, escapes further cytosolic destruction and is transported by ATP-binding cassette (ABC) transporters into the endoplasmic reticulum (ER) and lysosomes. The ER-resident heterodimeric transporter associated with antigen processing (TAP) is a crucial component in adaptive immunity for the transport and loading of peptides onto major histocompatibility complex class I (MHC I) molecules. Although the function of the lysosomal resident homodimeric TAPL-like (TAPL) remains, until today, only loosely defined, an involvement in immune defense is anticipated since it is highly expressed in dendritic cells and macrophages. Here, we compare the gene organization and the function of single domains of both peptide transporters. We highlight the structural organization, the modes of substrate binding and translocation as well as physiological functions of both organellar transporters.

## Introduction

**Figure 1:**
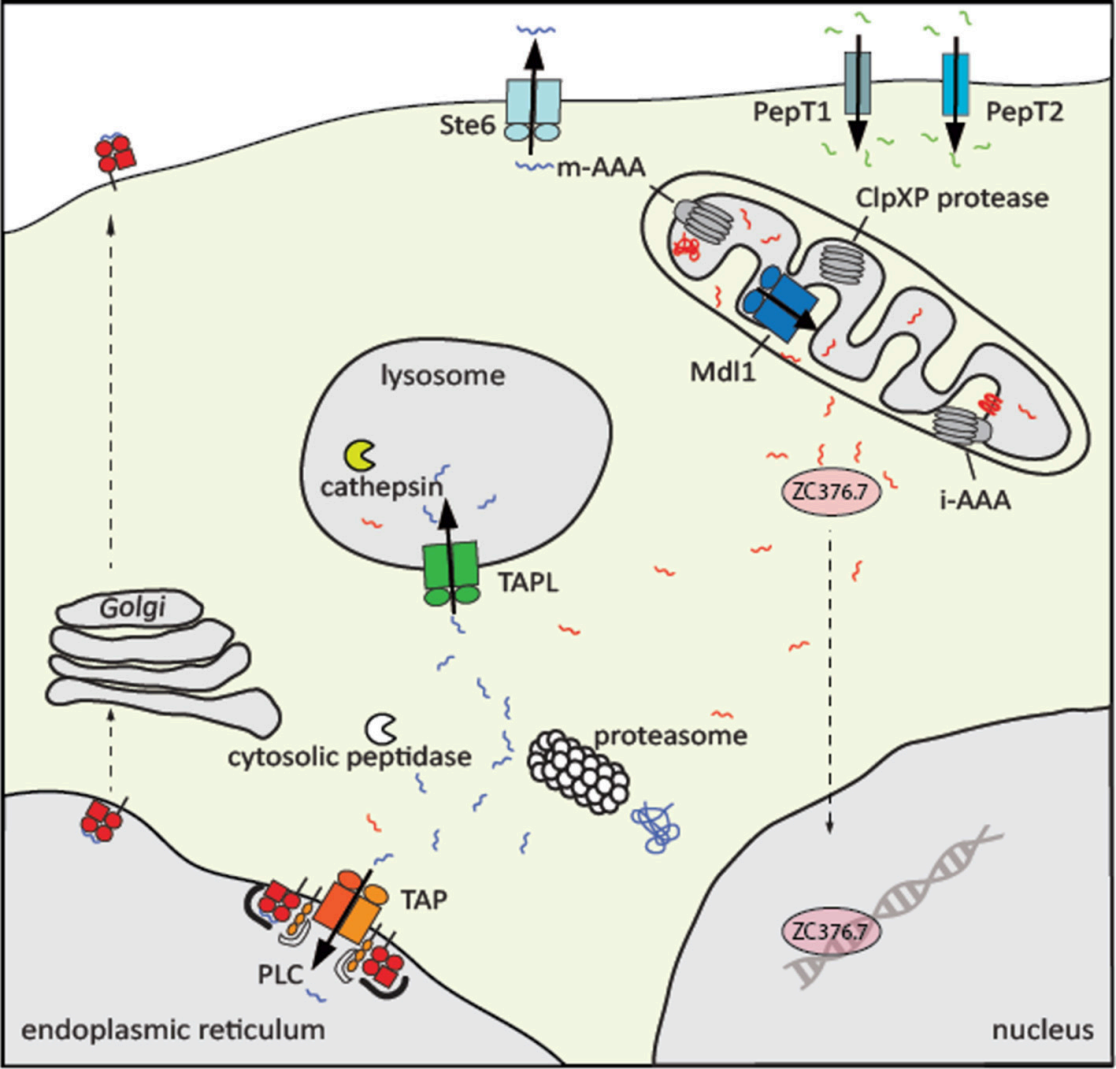
Physiological role of eukaryotic peptide transporters. Peptide transporters are localized at different cellular membranes. At the plasma membrane of intestine and kidney cells, the secondary active transporters PepT1 and PepT2 import di- to tetramer peptides along a proton gradient. All peptide transporters belonging to the ABC superfamily are exporters. The peptide pheromone exporter Ste6 from *Saccharomyces cerevisiae* shuttles the mating a-factor into the extracellular space. Proteins in the matrix and inner membrane of mitochondria are degraded by the AAA+ proteases m-AAA and ClpXP and the resulting peptides are transported by Mdl1 in yeast, HAF-1 in *C. elegans*, and probably ABCB10 in human into the intermembrane space. Subsequently, the transcription factorZC376.7 (Atfs1) in *C. elegans*, the homolog of Atf5 in human, is activated and localized in the nucleus, where genes of the mitochondrial unfolded protein response are induced. Cytosolic proteins are degraded mainly by the proteasome. Peptides that escape further trimming by cytosolic peptidases are translocated by the heterodimeric ABC transporter associated with the antigen processing TAP into the ER, where these peptides are loaded onto MHC class I molecules to present the antigenic peptides on the cell surface to cytotoxic T-cells. The peptide transfer, editing, and proofreading is catalyzed by the peptide loading complex (PLC). In addition, cytosolic peptides are transported by TAPL to lysosomes for further processing by cathepsins.

In the life cycle of a cell, the proteome is metastable and dynamically shaped by synthesis, folding, modification, and degradation. Proteins are degraded when either being damaged, matched for a demanded life-time, and no longer used, or delivered as defective ribosomal products. The clearance of these proteins starts in the cytosol with hydrolysis to peptides of three to twenty residues in length predominately through the ubiquitin-proteasome system (UPS). Malfunction of this macromolecular degradation machinery is associated with neurodegenerative, autoimmune, and rheumatoid diseases, viral infections, and cancer (Schmidt and Finley, 2014). Most of the peptides generated by the UPS are processed within seconds to amino acids via cytosolic oligo- and aminopeptidases (Reits et al., 2003). Some of these peptides escape degradation to fulfill important functions (Figure 1). A well-studied example is the mating a-factor functioning as pheromone in yeast. The precursor of 36 amino acid length encoded by *MFA1* undergoes six consecutive steps of post-translational modification, yielding a mature 12-residue long a-factor, which is expelled to the external medium by the ATP-binding cassette (ABC) transporter Ste6 (Michaelis and Barrowman, 2012). In *Caenorhabditis elegans* mitochondrial unfolded stress response is signaled to the nucleus via peptides. In this process, mitochondrial proteins are degraded by ATP-dependent degradation machine ClpXP in the matrix and peptides are released to the cytosol most likely by the ABC transporter HAF-1. Here, the peptides directly or indirectly bind to a transcription factor, which induces the expression of mitochondrial chaperones to cope with mitochondrial proteostasis (Haynes et al., 2010). The analysis of the mouse brain peptidome by mass spectrometry demonstrated that some intracellular peptides are enriched. It was therefore speculated that peptides can take over regulatory functions (Fricker, 2010).

The most well-studied and medically important function of cytosolic protein fragments is their role in building an adaptive immune response (Figure 1). Proteasomal degradation products are shuttled by the ABC transporter associated with antigen processing (TAP1/2, ABCB2/ABCB3) into the lumen of the endoplasmic reticulum (ER) where these peptides are loaded onto major histocompatibility complex class I (MHC I) molecules (Abele and Tampé, 2004; Parcej and Tampé, 2010; Blum et al., 2013). Peptide-MHC I complexes are subsequently transported to the cell surface in order to present the bits of the cellular proteome as metabolic snapshots to cytotoxic CD8^+^ T-lymphocytes. If the T-cell receptor recognizes antigenic “non-self” peptides in complex with MHC I as “self” component, virally or malignantly transformed cells will be destroyed (Gromme and Neefjes, 2002). Importantly, cytosolic peptides are also frequently found on MHC II molecules, which is an essential step in the negative selection during T-cell development to impede autoimmune response (Crotzer and Blum, 2010).

Different pathways seem to contribute to the delivery of cytosolic peptides to the lysosomes (Figure 1). Cytosolic proteins are typically delivered by macro-autophagy or by chaperone-mediated autophagy to lysosomes, where they are degraded by cathepsins (Crotzer and Blum, 2008). However, cytosolic peptides can also be transported directly into lysosomes and bound to MHC II without further processing. It was speculated that the transporter associated with antigen processing-like (TAPL, ABCB9) is involved in this process (Dani et al., 2004). Here, we summarized some structural and mechanistic similarities but also a number of remarkable differences of these TAP-related peptide transporters.

## The diversity of organellar peptide transporters

Different classes of peptide transporters have evolved (Figure 1). All in common is a high substrate promiscuity. Peptides containing two to eight residues are transported by members belonging to the oligopeptide transporter and peptide transporter family (Gomolplitinant and Saier, 2011; Newstead, 2015). Both families belong to the Major Facilitator Superfamily of secondary active transporters, which are proton-dependent transport systems. Oligopeptide transporters, translocating peptides of three to eight amino acids in length, are found in bacteria, plants, and fungi. Di- and tripeptide transporters are also present in animals. Human PepT1 and PepT2 are found in the brush border membrane of the small intestine and at the renal epithelium in the kidney, respectively. Both transporters absorb or retain protein fragments in the body. Interestingly, PepT1 is the fast, low-affinity transporter while PepT2 shows slower transport rates paired with higher affinity (Brandsch, 2013).

Longer peptides are typically handled by ABC transporters, the largest family of primary active transporters. In bacteria, oligopeptides are complexed in the periplasm by a specific binding protein, which hands over the substrate to an ABC import system (Doeven et al., 2005). In eukaryotes, only oligopeptide ABC exporters are described. A vacuolar ATP dependent transporter was identified in plants, translocating peptides into the lumen of vacuoles (Ramos et al., 2011). Furthermore, peptide transporters with different intracellular localization exist in yeast, nematodes, and vertebrates (Herget and Tampé, 2007). In yeast, Mdl1 is located in the inner mitochondrial membrane and is proposed to transport degradation products of matrix or inner mitochondrial membrane proteins into the intermembrane space (Young et al., 2001).

**Figure 2:**
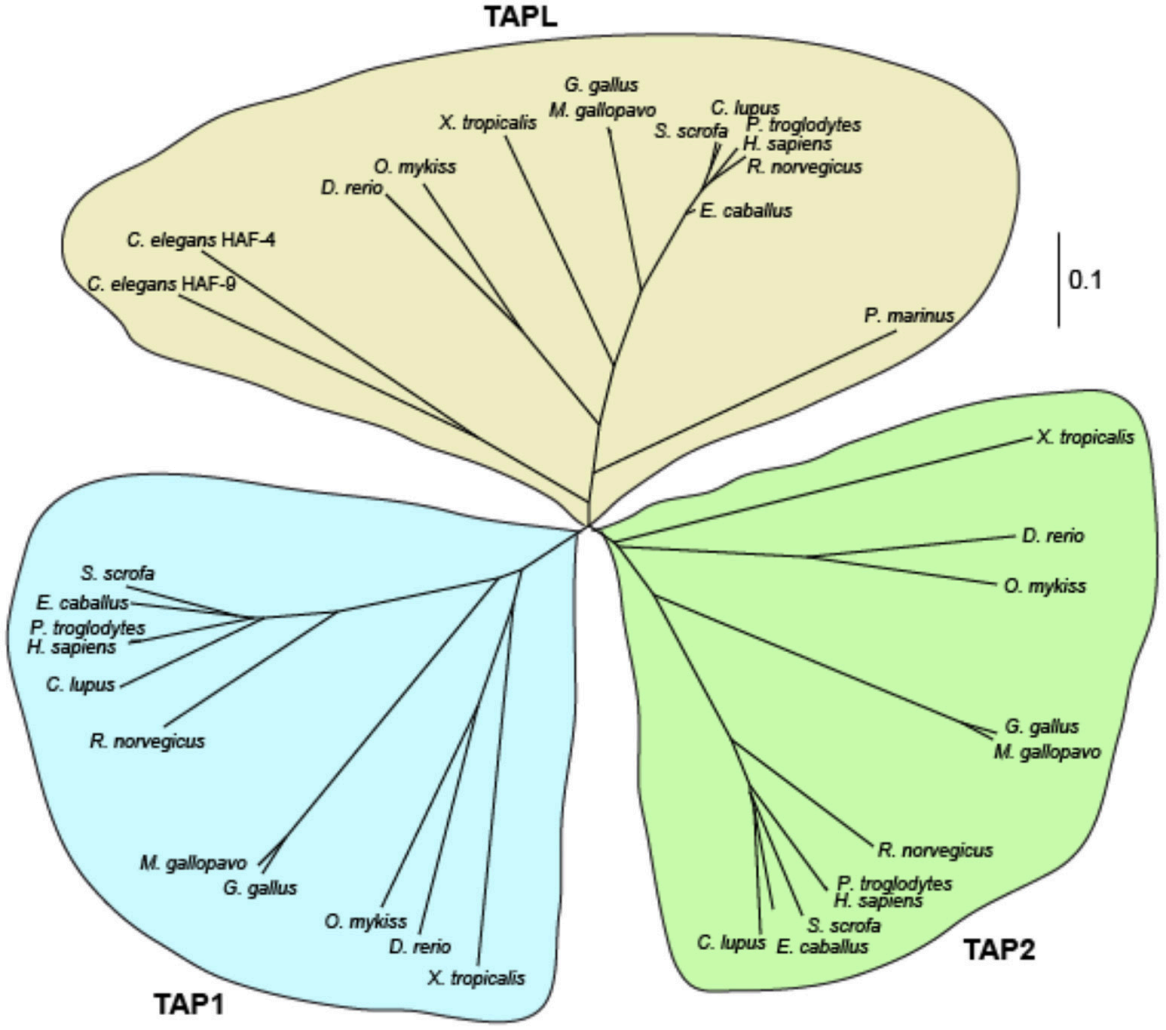
Phylogenetic relationship of TAP and TAPL. Multiple sequence alignment of TAPL, TAP1, and TAP2 variants of human (*Homo sapiens*), chimpanzee (*Pan troglodytes*), horse (*Equus cabalus*), pig (*Sus scrofa*), dog (*Canis lupus*), rat (*Rattus norvegicus*), turkey (*Meleagris gallopavo*), chicken (*Gallus gallus*), trout (*Oncorhynchus mykiss*), zebrafisch (*Danio rerio*), western clawed frog (*Xenopus tropicalis*), sea lamprey (*Petromyzon marinus*), and roundworm (*Caenorhabditis elegans*) were performed with Clustal Omega (Sievers et al., 2011). An unrooted cladogram was drawn by Phylodendron (http://iubio.bio.indiana.edu/treeapp/). The bar indicates the evolutionary change rate. In the lower vertebrate lamprey and in the nematode *C. elegans* only homologs of TAPL are found. Longer branches of mammal and avian homologs reflect the higher evolutionary rate of TAP1 and TAP2 in comparison to TAPL.

The TAP family is composed of three half-transporters, TAP1 (ABCB2), TAP2 (ABCB3), and TAPL (ABCB9). Members of the TAP family are also found in *C. elegans* and in chordata but not in insects and crustaceans (Figure 2). TAP1 and TAP2 must pair to constitute a transport-competent complex, whereas TAPL forms homodimers (Powis et al., 1991; Leveson-Gower et al., 2004). Interestingly, HAF-4 and HAF-9 of *C. elegans* are orthologs of TAPL with a sequence identity of 38%. Both half-transporters are localized in large granules of intestinal cells. Loss-of-function mutants of either of these genes result in loss of these large non-acidic granules, accompanied by decreased brood size, extended defecation cycle, and slow growth (Kawai et al., 2009). HAF-4 and HAF-9 appear to form heterodimers and active heterodimers are essential for granule formation (Tanji et al., 2013).

In contrast to jawed vertebrates, which express TAP1, TAP2, and TAPL, only one member belonging to the TAP family was detected in agnatha (jawless vertebrates) and even in tunicates (Uinuk-ool et al., 2003; Ren et al., 2015). The half-transporter from the jawless vertebrate sea lamprey shows a higher sequence identity to human TAPL (52.4%) than to TAP1 (38.4%) and TAP2 (40.7%). Therefore, ABCB9 from agnatha can be regarded as the progenitor of the TAP family. Interestingly, TAP1 and TAP2 have evolved much faster than TAPL. Comparing the amino acid sequences from rat and mouse, the replacement rates in TAP1 and TAP2 are more than 10 times faster as in TAPL (Kobayashi et al., 2000).

In mammals, TAP and TAPL show a broad tissue distribution. TAP was found in nearly every nucleated cell. TAPL is also detected in all tissues examined (Bgee database: Bastian et al., 2008; The Human Protein Atlas: Uhlén et al., 2015) with high expression in the central nervous system and testis (Yamaguchi et al., 1999; Zhang et al., 2000; Mutch et al., 2004). Interestingly, TAPL is not found in monocytes but highly expressed in dendritic cells and macrophages implying a role in the immune response (Demirel et al., 2007).

## Physiological function

Although TAP and TAPL transport a similar range of peptides, their physiological functions are largely different. In the past, the paradigm was that MHC I presents peptides from endogenous antigens while MHC II displays peptides from exogenous antigens. Through analyzing the peptidome of MHC molecules it became evident that this sharp border is not correct. A quantitative amount of MHC II molecules is loaded with peptides from cytosolic origin (Stern and Santambrogio, 2016). Moreover, in professional antigen presenting cells a process called cross-presentation takes place, in which peptides of exogenous antigens are loaded on MHC I molecules (van Endert, 2016; Grotzke et al., 2017b). This pathway ensures the induction of an adaptive immune response to tumor antigens and antigens derived from pathogens, which does not infect dendritic cells. TAP is an essential machinery in delivering cytosolic peptides, mainly produced by the UPS, into the lumen of the ER for loading onto MHC I. Importantly, TAP is not only crucial for the classical MHC I presentation but also actively involved in the cross-presentation pathway. In the TAP-dependent cross-presentation, exogenous antigens are taken up by phagocytosis or receptor-mediated endocytosis. Subsequently, the antigen is transported from the phagosome or endosome into the cytosol putatively by Sec61 (Koopmann et al., 2000; Zehner et al., 2015; Grotzke et al., 2017a). In the cytosol, the antigen is proteasomally degraded and peptides are transported by TAP into the ER or phagosome/endosome for loading onto MHC I.

The importance of TAP in antigen presentation is highlighted in the Bare Lymphocyte Syndrome type I (BLS-I), a rare disease caused by TAP deficiency (Zimmer et al., 1998). Cells of BLS-I patients show a strong decreased cell surface expression of MHC I accompanied by a reduced number of CD8^+^ T-cells. BLS-I patients do not suffer from a prevalence of viral infections but instead show chronic necrotizing lesions in the lung and skin escorted by recurrent bacterial infections. Moreover, the key function of TAP is emphasized by the armada of viral factors inhibiting TAP (Mayerhofer and Tampé, 2015; van de Weijer et al., 2015). These viral immune evasins all derive from large DNA viruses belonging to the family of Herpesviridae or Poxviridae. Each viral factor has its own inhibition mechanism dealing with peptide binding, ATP binding, conformational changes, or proteasomal degradation of TAP (Mayerhofer and Tampé, 2015).

In addition to the effects on the formation of gut granules in *C. elegans*, the physiological function of TAPL remains loosely defined. Since TAPL is found in nearly all tissues, a housekeeping function can be assumed to protect the cytosol from accumulation of otherwise harmful peptides. However, TAPL may have a more specialized function in professional antigen-presenting cells, in which its expression is strongly upregulated (Demirel et al., 2007). There are alternative pathways of endogenous antigens for processing and loading on MHC II (Veerappan Ganesan and Eisenlohr, 2017). One of these pathways is proteasome dependent but TAP-independent (Dani et al., 2004; Miller et al., 2015; Thiele et al., 2015). Furthermore, the loading of MHC II occurs in the lysosomal compartment (Dani et al., 2004). Therefore, it can be speculated that TAPL is the translocation machinery in this process, which is supported by the interaction of TAPL with MHC II (Demirel et al., 2012).

There are several cross-presentation pathways for presenting exogenous antigens on MHC I. The main difference consists in the subcellular compartment of antigen degradation. In the vacuolar pathway, the endocytosed or phagocytosed antigen is transported to the endolysosomal system where the antigen is digested and loaded onto MHC I molecules. In the cytosolic pathway, the antigen is translocated to the cytosol for proteasomal degradation. Subsequently, antigenic peptides are transported into the ER or into phagosomes for MHC I loading. Although TAP is the main transport complex in this pathway, it was recently shown in mouse dendritic cells that a TAP-independent but ATP-consuming machinery exists for peptide loading of phagosomes (Merzougui et al., 2011; Lawand et al., 2016). Although the phagosomal localization and the peptide specificity of mouse TAPL still has to be evaluated, TAPL could possibly be this uncharacterized peptide transporter.

## Gene organization

The genes coding for human TAP1 and TAP2 are localized in the MHC II locus of chromosome 6 and their expression is induced by IFN-γ (Deverson et al., 1990; Monaco et al., 1990; Spies et al., 1990; Trowsdale et al., 1990, 1991). In contrast, the *tapl* gene is found on chromosome 12, lacking any link to factors of the adaptive immune system. In addition, *tapl* expression is not affected by IFN-γ (Kobayashi et al., 2000). All three genes are composed of 11 coding exons whereas *tapl* and *tap2* possess an additional 5′-non-coding exon. Besides the flanking coding exons, the exon length of all three genes is identical with the exception of coding exon 9 with identical length in *tap2* and *tapl* but 3 bp elongated in *tap1*. Strikingly, the length of the introns is much longer for *tapl* than for *tap1* and *tap2* (Uinuk-ool et al., 2003). For TAP1 only one splice isoform has been reported. However, a short (748 amino acids, aa) and a long (808 aa) variant of human TAP1 is found in the database, probably caused by alternative translation start sites. TAP2 has two splice isoforms of 653 and 703 aa with different 3′-terminal exons and varying peptide specificity (Yan et al., 1999). In addition to several allelic variants with single amino acid substitutions, a short and a long allelic variant of TAP2 with 686 and 703 aa has been described (Colonna et al., 1992). However, the expression of these different TAP1 and TAP2 variants and the impact on antigen presentation is not studied in detail.

For TAPL, six splice isoforms have been assigned in the UniProt database. The splice variants 12A, 12B, and 12C differ in the 3′-terminal exon (Kobayashi et al., 2003). Isoform 12B and 12C are likely inactive in peptide transport since both variants lack the conserved H-loop being essential for ATP hydrolysis. Two additional isoforms are probably non-functional as they lack the coding exon 6 or 7, which comprises transmembrane helices (TM) 9 and 10, and the sequence connecting the transmembrane domain (TMD) with the nucleotide-binding domain (NBD) (Zhang et al., 2000). The sixth isoform of TAPL is characterized by the absence of almost the entire NBD. Biochemical and cell biological data are available only for isoform 12A, the longest reported version with 766 aa. The activity and physiological function of the other TAPL variants is not resolved and questionable.

## Domain organization of TAP-related transporters

**Figure 3:**
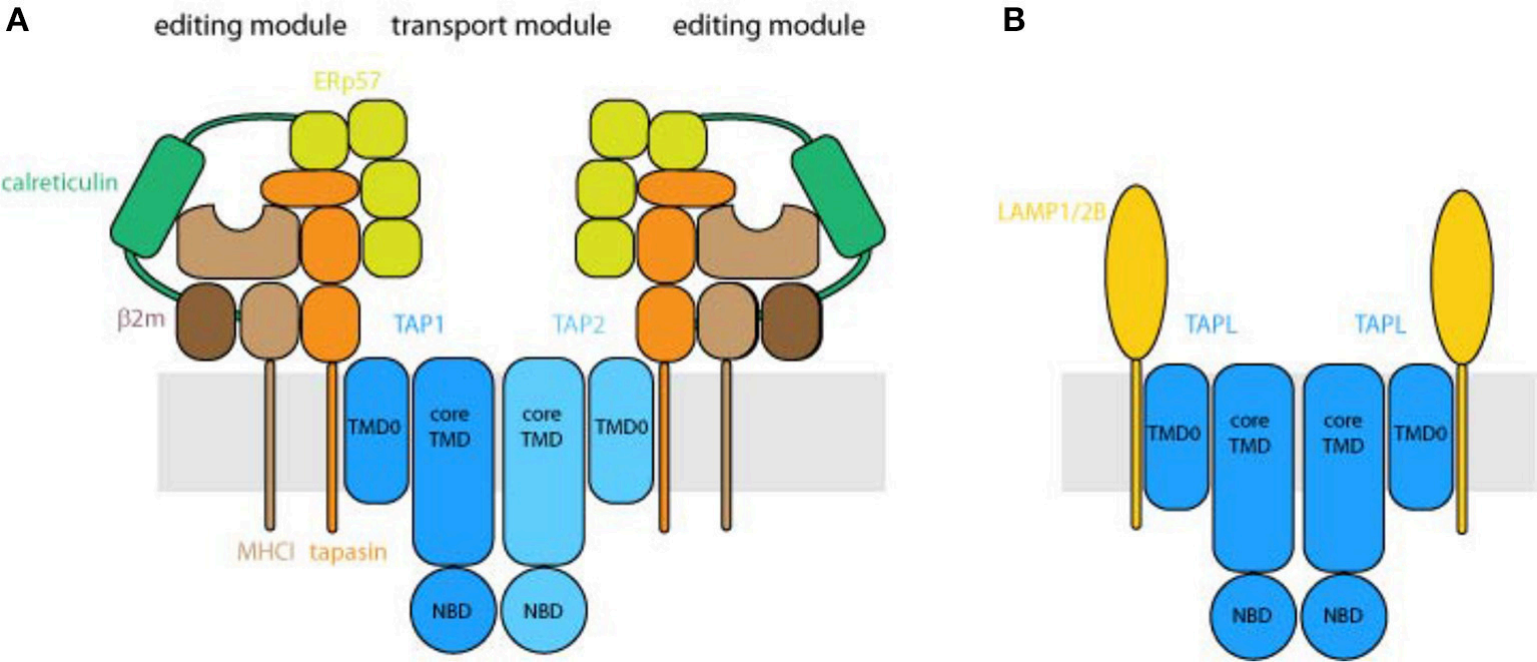
Organization of the peptide transport machineries. **(A)** Architecture of the peptide loading complex. TAP composed of the half-transporter TAP1 and TAP2 forms the central translocation unit of the peptide loading complex. Each half-transporter consists of an N-terminal transmembrane domain (TMD) followed by a cytosolic nucleotide-binding domain (NBD). The core transporter, formed by the core TMDs, composed of 2 × 6 transmembrane helices and two NBDs, is fully active in peptide transport. The N-terminal TMD0s of TAP1 and TAP2, harboring four transmembrane helices each, interact with the transmembrane helix of tapasin. As central piece of the editing module, tapasin binds to ERp57, calreticulin and MHC I and destabilizes peptide MHC I interaction that only MHC I molecules loaded with high affinity peptides can shuttle to the cell surface. Depicted are two fully assembled editing modules, which can be found only transiently in cells. **(B)** TAPL forms a homodimeric transporter whereas the core transporter is composed of the 2 × 6 transmembrane helices and two NBDs. The four transmembrane helices comprising N-terminal TMD0 mediates lysosomal targeting as well as the interaction with LAMP1/2B. The stoichiometry between TAPL and LAMP1/2B is not solved.

All ABC transporters are modularly organized and consist of two NBDs and two TMDs. These domains are arranged either in the form of four polypeptides, one polypeptide, or as a fusion of two domains. In eukaryotes, only full transporters with all four domains in one polypeptide chain, or half-transporters composed of one TMD and one NBD, exist (Locher, 2016; Thomas and Tampé, 2018). TAP1, TAP2, and TAPL are half-transporters. TAPL forms a symmetric homodimer whereas TAP is composed of TAP1 and TAP2 and thus asymmetric (Powis et al., 1991; Leveson-Gower et al., 2004) (Figure 3). In addition to their conserved four-domain-architecture, termed coreTAP and coreTAPL, each transporter subunit carries an extra N-terminal four-transmembrane helix domain named TMD0. For both complexes, the TMD0 is not required for peptide transport (Koch et al., 2004; Demirel et al., 2010). However, the TMD0s mediate the direct interaction with type I membrane proteins in different cellular compartments.

The TMD0s of TAP1 and TAP2 are essential for the assembly of the MHC I peptide-loading complex (PLC), composed of TAP1, TAP2, tapasin, MHC I, calreticulin, and ERp57, and consequently for efficient loading of MHC I molecules with antigenic peptides (Hulpke et al., 2012b) (Figure 3A). As illuminated by the cryo-EM structure of the human PLC, TAP as transport module is encircled by two editing modules (Blees et al., 2017). Tapasin as central factor of the editing module binds to MHC I, ERp57, and calreticulin, which spans as a clamp around the editing module and therefore seems to stabilize the subcomplex. Interestingly, the two editing modules are scaffolded by two tapasin molecules, which are stacked to each other via salt bridges. Tapasin comprises the editing function guaranteeing that only MHC I molecules loaded with stably bound, high-affinity peptides can leave the ER and are shuttled to the cell surface (Blees et al., 2017). The TMD0s of TAP1 and TAP2 interact independently from each other with the transmembrane helix of the MHC I specific chaperone tapasin (Hulpke et al., 2012a). For human TAP, the interaction between TAP and tapasin is mainly mediated by a salt bridge in the hydrophobic core of the ER membrane between a conserved aspartate in TM1 of each TAP subunit and a lysine of tapasin (Blees et al., 2015). Notably, an exchange of the conserved aspartate to lysine in TM1 of TAP1 or TAP2 abolished the PLC assembly, which was rescued by a double lysine-to-aspartate exchange. For rat TAP, however, this conserved aspartate in TAP2 can be exchanged to alanine without an effect on tapasin binding. In this case, leucine-rich areas in TM1 and TM2 of TAP2 seem to be important for tapasin binding (Rufer et al., 2015). Interestingly, tapasin interacts also with core TM9 of unassembled TAP1 which facilitates transporter stability and heterodimerization (Leonhardt et al., 2014). Hence tapasin may act as a dummy protein as found in the pre-B-cell receptor to guarantee correct folding of TAP1 which is a prerequisite for TAP2 assembly (Keusekotten et al., 2006).

In the case of TAPL, the TMD0 interacts with the lysosomal associated membrane proteins LAMP-1 and LAMP-2B but not with the splice variant LAMP-2A, which is the receptor for chaperone-mediated autophagy (Figure 3B). Remarkably, the interaction with LAMP-1 protects TAPL from lysosomal degradation and significantly increases its half-life (Demirel et al., 2012). Furthermore, the TMD0 of TAPL is essential and sufficient for lysosomal targeting of the complex. Solely expressed TMD0 is transported to lysosomes while coreTAPL traffics to the plasma membrane. Coexpression of both modules leads to their stable association and lysosomal localization (Demirel et al., 2010). The interaction with LAMP-1/2 has an impact neither on the lysosomal localization nor on the transport activity of TAPL (Demirel et al., 2012). Since TMD0 does not contain one of the conventional di-leucine or acidic-based lysosomal targeting motifs (Bonifacino and Traub, 2003), the targeting mechanism is currently unknown. In contrast, TAP retention in the ER is independent of its TMD0s. Unlike TAPL, coreTAP does not interact with the TMD0s (unpublished results).

## Cryo-EM and X-ray structures of TAP-related transport systems

**Figure 4:**
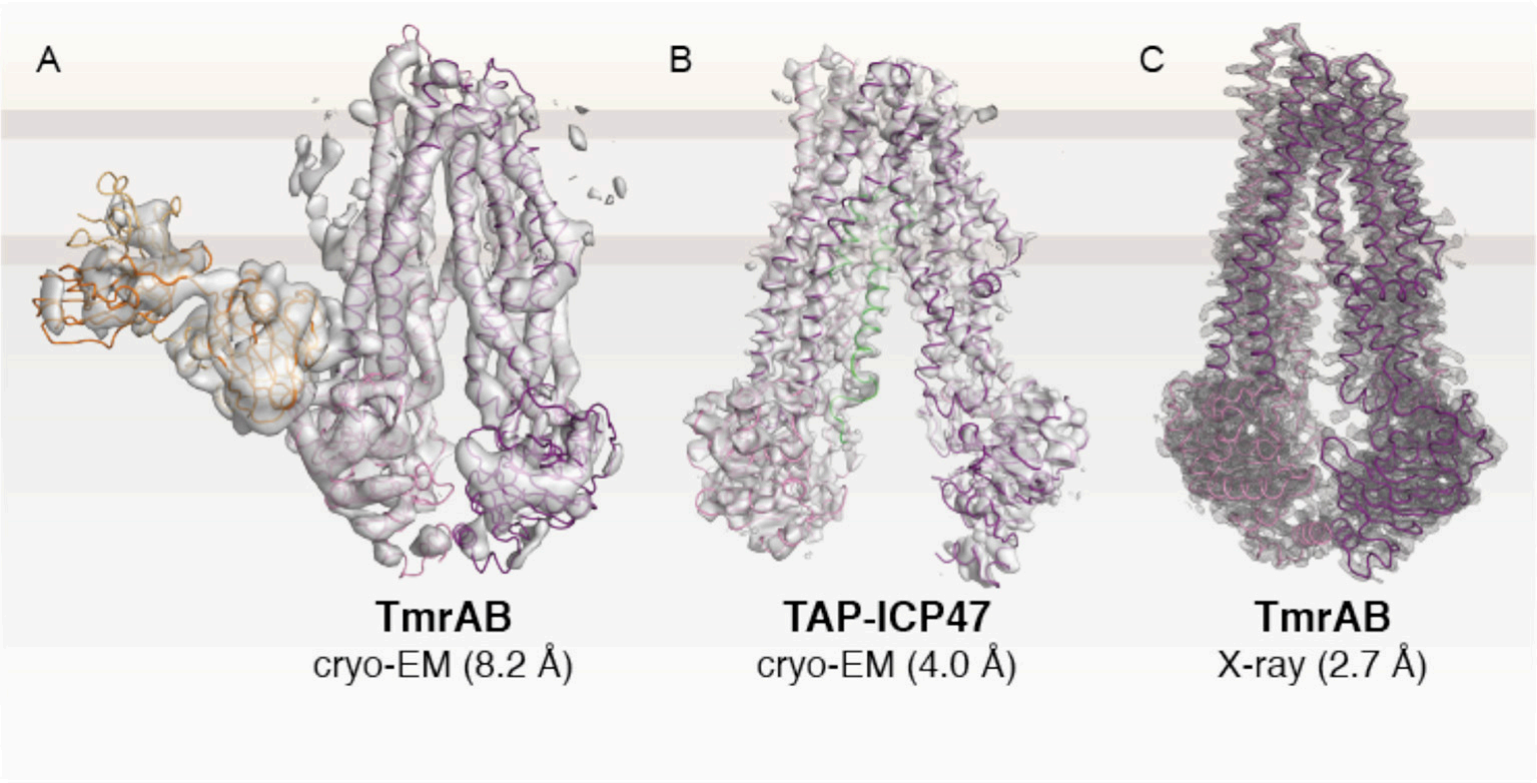
Structures of TAP-related peptide transporters. **(A)** First high-resolution cryo-EM structure of an ABC transporter (Kim et al., 2015). The heterodimer TmrAB, a functional homolog of the TAP complex, was determined in an apo, inward-facing conformation in complex with a specific antibody fragment (EMD 6085. **(B)** Cryo-EM structure of human TAP in complex with ICP47 (EMD 8482; PDB 5U1D) at 4.0 Å (Oldham et al., 2016a). The N-terminal TMD0 of TAP1 and TAP2 and the C-terminal part of ICP47 are not visible due to their high flexibility. **(C)** X-ray structure of TmrAB at 2.7 Å resolution (PDB 5MKK) (Nöll et al., 2017). TmrAB and TAP1/2 are illustrated in light and dark magenta. The Fab fragment and ICP47 as fiducial marker or stabilizing factor are colored in yellow or green, respectively. The membrane border is indicated by thick gray lines.

In recent years, the ABC transporter field has experienced a revolution after reporting on the first high-resolution cryo-EM structure of an ABC transporter (Figure 4A) (Kim et al., 2015). The heterodimeric transporter TmrAB from *Thermus thermophilus* was selected from a structural genomic screen of TAP-related transporters. TmrAB was identified as a functional homolog of human TAP, complementing antigen processing and presentation in TAP-deficient cells isolated from BLS-I patients (Nöll et al., 2017). Despite the small complex size of 135 kDa and its pseudo-symmetric organization, cryo-EM revealed new insights into the asymmetric organization of TAP-related ABC transporters. Simultaneously, the X-ray structure of TmrAB in an apo, inward-facing conformation was determined at 2.7 Å resolution, providing atomistic details of peptide translocation complexes (Nöll et al., 2017) (Figure 4C).

The cryo-EM studies on TAP-related translocation complex TmrAB paved the way for numerous subsequent cryo-EM studies on ABC transporters, including human TAP (Oldham et al., 2016a,b), bovine MRP1 (Johnson and Chen, 2017, 2018), zebrafish and human CFTR (Liu et al., 2017; Zhang et al., 2017), ABCA1 (Qian et al., 2017), ABCG2 (Taylor et al., 2017), and SUR1 (Li et al., 2017; Martin et al., 2017a,b). To improve the resolution by cryo-EM, human TAP was arrested in a single conformation by the viral inhibitor ICP47, which has previously been shown to bind with high affinity to TAP (Ahn et al., 1996; Tomazin et al., 1996). By optimizing cryo-EM imaging and data processing, an average resolution of 4.0 Å was achieved in the core transport complex (Figure 4B), with higher resolution in TMDs, sufficient for registration of side chains, but at lower resolution in the NBDs, allowing the assignment of polypeptide backbone only (Oldham et al., 2016a). Due to their high flexibility, the TMD0s and the C-terminal part of the viral inhibitor comprising one-third of the complex could not be resolved.

The core translocation unit of TAP and also TmrAB resemble the structure of an inward-facing type I ABC exporter with 12 TMs where TM1-3 and 6 of one subunit and TM4/5 of the other subunit constitute one wing of the TMDs (Kim et al., 2015; Oldham et al., 2016a,b; Nöll et al., 2017). The NBDs are separated from each other but connected to the TMDs by cytosolic loops. In this ICP47-inhibited state, the interaction of the cytosolic loops is restricted to one NBD (ICL1 intra- and ICL2 intermolecular) and not both NBDs, as observed by disulfide crosslinking of TAP in ER membranes, when several conformations can be sampled (Oancea et al., 2009). The resolution of the NBDs, however, is not sufficient to allow detailed insights in the asymmetry of both TAP subunits. The first 55 residues of ICP47 form an α-helical hairpin structure and contact mainly TMs from TAP2 with an interface twice the average size of a binding interface, explaining the strong interaction and thermostability of the TAP complex (Herbring et al., 2016). The borders of the helical elements show a small deviation from the NMR structure of the active domain of ICP47 (residue 2–34) (Pfänder et al., 1999; Aisenbrey et al., 2006). The active domain has the same affinity as full-length ICP47 to inhibit peptide binding and transport, although the additional 21 residues resolved in the cryo-EM structure form close interactions with TM3 and cytosolic helix 1 of TAP2 (Galocha et al., 1997; Neumann et al., 1997). Although ICP47 interacts with TAP differently than antigenic peptides, the binding sites for the competitive inhibitor ICP47 and the peptides overlap at least partially, derived from a comparison of the ICP47-bound structure with biochemical data (Lehnert and Tampé, 2017). Remarkably, the inward-facing conformation of the ICP47-arrested TAP shows some distortion in the transmembrane region if compared to the X-ray structure of the TAP ortholog TmrAB. The lumenal gate is not closed, most likely induced by ICP47 binding, which leads to a strong bending of TM4/5 of TAP1 reflecting an altered cross-linking behavior (Lacaille and Androlewicz, 1998).

## Peptide binding

As for most ABC transporters, high-resolution structures with a bound substrate are still missing. Presently, the peptide-binding site of TAPL is not well-characterized because of the apparent micromolar peptide affinity (Table 1) (Wolters et al., 2005; Zhao et al., 2008). For TAP, molecular docking of peptides to homology models of TAP was performed to elucidate the peptide-binding site (Corradi et al., 2012; Geng et al., 2015; Lehnert et al., 2016). In all three studies, the binding site is localized in the transmembrane region. Depending on the template, and therefore on the opening of the TMD used to build the homology model of TAP, on the procedure to restrain the conformation of the peptide and on the method to dock the peptide, the conformation of the bound peptide as well as the exact localization within the TMD differ greatly. TAP inhibited by ICP47 is not the appropriate structure for docking experiments, since it shows obvious deviations in the TMD from other ABC transporters (see above). The conformation of the peptide has the strongest impact on the docking. Since, up to now, it has not been possible to dock a 9-mer peptide to the large cavity of TAP because of the high degree of freedom, all three studies had to cope with constraints concerning the peptide conformation. One study performed replica exchange simulations, in which the C_α_ of the N-terminal and C-terminal residue of the peptide were restrained to positively and negatively charged pockets assigned as binding sites for the N-terminal amino and the C-terminal carboxy group (Corradi et al., 2012). The peptide adopts an extended conformation. A second approach restrained the peptide in a β-hairpin conformation, which allowed docking in a position matching their crosslinking data (Geng et al., 2015).

**Table 1:** Characteristics of TAP-related transporters.

	**TAP1/2**	**TAPL**
Localization	ER (phagosomes)^#^ (endosomes)^#^	Lysosomes (non-acidic granules)
Core transport unit	Heterodimer (6 + 6 TMs)	Homodimer (6 + 6 TMs)
Interaction module	TMD0 (4 TMs)	TMD0 (4 TMs)
Interaction partner	TAPBR (tapasin)	LAMP-1, LAMP-2B
ATP-binding sites	Asymmetric	Symmetric
ATPase activity	Strictly coupled	Uncoupled
Substrate specificity	Peptides (8–16 aa) position 1-3 and Ω	Peptides (6–59 aa) position 1 and Ω
Peptide affinity	High-affinity (50 nM)	Low-affinity^*^ (10 μM)
Trans-inhibition	Yes (16 μM)	Yes (1 mM)
Viral inhibitors	ICP47, US6, UL49.5, BNLF2a, CPXV012	

# *Antigen cross-presentation*;

* *derived from K_m_-value*.

Recently, the backbone structure of TAP-bound peptides was determined by dynamic nuclear polarization enhanced magic angle spinning solid-state NMR and subsequently used for molecular docking (Lehnert et al., 2016). This experimentally determined peptide structure adopts an extended conformation and perfectly agrees with pulsed EPR data, which showed for a similar peptide a distance of 2.5 nm of both termini (Herget et al., 2011). In the models by Corradi et al. and Lehnert et al. the binding pockets for the N- and C-terminus of the peptide are separated by 2.5 nm (Herget et al., 2011; Corradi et al., 2012; Lehnert et al., 2016; Lehnert and Tampé, 2017). The N-terminus of the peptide binds in both models to the same negatively charged pocket. However, in the model presented by Corradi et al. the peptide is aligned parallel to the membrane plane, whereas in the model of Lehnert et al. the peptide is more perpendicular to the membrane plane. Therefore, the C-termini of the extended peptides bind to different positively charged pockets. In the bent peptide structure modeled by Geng et al. there are no charged pockets within TAP involved in the binding of the N- and C-terminal group since they form a salt bridge by themselves (Geng et al., 2015). While the distance of the charged binding pockets perfectly fits with the minimal peptide length recognized by TAP, the binding site in the model of Geng et al. cannot explain the size restriction.

## Specificity of peptide transporters

TAP and TAPL are polypeptide ABC exporters, which move peptides out of the cytosol into the ER or into lysosomes, respectively. TAP prefers 8–16-mer peptides whereas TAPL displays a broader length window from 6 to 59-mer peptides with an optimum for 23-mer peptides (van Endert et al., 1994; Uebel et al., 1997; Wolters et al., 2005). The N-terminal amino group as well as the C-terminal carboxy group are involved in peptide binding since modifications of the termini interrupt peptide binding and transport. In initial experiments, using peptides composed of D-amino acids, the importance of side chains for substrate specificity was recognized. With combinatorial peptide libraries, the sequence specificity of both transporters was evaluated (Uebel et al., 1997; Zhao et al., 2008). For TAP, the first three N-terminal and the last C-terminal residues are relevant for peptide selection, whereas TAPL senses only the terminal residue on both ends. The selectivity pattern for TAP and TAPL is related: both favor basic and bulky residues and disfavor negatively charged side chains at their termini (Uebel et al., 1997; Zhao et al., 2008). Furthermore, for TAP a proline at position two and acidic residues at position three strongly interfere with peptide binding. Since peptides with a proline are found at position two on the peptide-recipient MHC I molecule, N-terminal trimming of the translocated peptides by ER-luminal peptidase ERAP-1 and-2 must occur (for review see van Endert, 2011). The sequence between both ends can be highly promiscuous for both transporters, and even large fluorophores or ε-amino linked polylysine chains with a mass equivalent to the unmodified peptide are tolerated at these positions (Grommé et al., 1997; Neumann and Tampé, 1999; Gorbulev et al., 2001). Although the peptide binding site seems to be large and flexible, TAP binds only one peptide as determined by fluorescence correlation spectroscopy (Herget et al., 2009). Whether TAPL as a homodimeric and therefore symmetric transporter binds more than one peptide is an open issue, however from existing transport studies any cooperativity in peptide binding can be excluded (Wolters et al., 2005; Zhao et al., 2008). The epitope RRYQKSTEL derived from histone 3.3 and fluorescently labeled derivatives were intensively used to study peptide binding and transport of TAP and TAPL. Based on its sequence, this peptide represents a good binder for both transporters. Interestingly, TAP binds this peptide with a 100-fold higher affinity as TAPL (Table 1). Peptide binding to TAP is a two-step process with a fast high-affinity association followed by a slow structural rearrangement of the transporter. Bound to TAP, the termini of the peptide feature a distance of 2.5 nm as revealed by pulsed EPR spectroscopy (Herget et al., 2011). This distance limits the size of the peptide to 8-mer peptides, which bind in an extended conformation discovered by solid-state NMR (Lehnert et al., 2016). Since longer peptides are restricted to the same distance, they have to adopt an extended kinked conformation in the binding pocket (Herget et al., 2011). Remarkably, the distance of the termini of MHC I bound peptides fits to that of TAP. It seems that not only in respect to sequence but also concerning the N- to C-terminal distance, MHC I and TAP has co-evolved and therefore TAP functions as a filter for MHC I. Taken together, TAPL recognizes a broader spectrum of peptides than TAP in respect to peptide length and sequence implying a more general function of the lysosomal transporter, respectively.

## Peptide translocation

TAP and TAPL export similar peptides out of the cytosol. Peptide transport is coupled with ATP hydrolysis since the non-hydrolysable ATP analog AMPPNP or mutations in the conserved sequences of the ATP binding site interfere with peptide translocation (Lapinski et al., 2001; Chen et al., 2004; Wolters et al., 2005; Perria et al., 2006; Demirel et al., 2007). Interestingly, mutation of the highly conserved aspartate of the D-loop of TAP1 to alanine turns TAP into a ligand-gated passive facilitator (Grossmann et al., 2014). The D-loop is localized in the interface between both NBDs and seems to be involved in communication between both ATP-binding sites. The ATP-binding site I of TAP formed by the Walker A/B motif of TAP1 and the C-loop of TAP2 shows strong deviation from the consensus sequences. This “degenerate” site has a strongly reduced ATP hydrolysis activity compared to the consensus site and is assumed to have a regulatory function not being directly involved in the energetics of peptide translocation (Lapinski et al., 2001; Chen et al., 2003). Interestingly, substituting the degenerate sequences by the consensus sequences creates a hyperactive transporter (Chen et al., 2004). TAP notably shows a direct coupling between peptide transport and ATP hydrolysis with no significant basal ATPase activity (Gorbulev et al., 2001; Grossmann et al., 2014). Moreover, there is a quality sensor since peptides, which bind to TAP but are not transported, do not stimulate ATPase activity (Gorbulev et al., 2001). Since TAPL has a high basal ATPase activity, a strong coupling between peptide binding, transport, and ATP hydrolysis is not expected (Zollmann et al., 2015).

Both transporters pump peptides against their concentration gradient as demonstrated by different techniques. Remarkably, TAPL accumulates peptides to an approximately 50-fold higher concentration than TAP. Both transporters do not reach the thermodynamic limit since they are inhibited in *trans*. This *trans* inhibition is comparable with product inhibition in classical enzyme kinetics. In the trans-inhibited state the transported peptide forces the transporter in the outward-facing conformation and therefore inhibits the conformational change to the inward-facing ground state. Subsequently, peptide transport and ATP hydrolysis are suppressed (Grossmann et al., 2014; Zollmann et al., 2015). The physiological meaning of this inhibition is an interesting point of discussion. In lysosomes, the peptides will be immediately degraded by the lysosomal proteases while in ER, the peptides will be bound to MHC class I and therefore removed from the pool of free peptides, or exported into the cytosol by an unresolved mechanism (Koopmann et al., 2000). The turnover rates of TAP and TAPL for peptide transport (*k*_cat_) determined in proteoliposomes by classical ensemble experiments, averaging over all molecules, indicate a low transport rate with a *k*_cat_ of approximately 0.1 peptide/min (Zhao et al., 2008; Schölz et al., 2011; Grossmann et al., 2014). The amount of functional purified and reconstituted transporters can vary immensely and cannot be determined correctly by these ensemble experiments. Therefore, the single-molecule based method dual color fluorescence burst analysis was applied on TAPL transport (Zollmann et al., 2015), by which the accumulation of fluorescently labeled peptides in single liposomes is monitored in the confocal volume of a microscope. Thereby, active transporters can be separated from inactive ones. Remarkably, only approximately 10% of TAPL was active in transport after reconstitution. To the end, a transport rate of eight peptides per min was determined, which fits to ATP hydrolysis kinetics of other well-characterized ABC exporters such as MsbA and ABCC3 (Zehnpfennig et al., 2009; Kawai et al., 2011). It is worth mentioning that data on solute transport kinetics are limited since most of the eukaryotic ABC exporters translocate hydrophobic substances, which are intrinsically problematic to analyze. Furthermore, TAP as well as TAPL operate strictly unidirectional and the process is not reversible as for the F_0_F_1_ ATP-synthase, which functions as ATP hydrolase or synthase depending on the proton gradient (Grossmann et al., 2014; Zollmann et al., 2015).

## Purification and functional reconstitution

A prerequisite for the detailed analysis of these transporters is their synthesis, purification, and functional reconstitution into liposomes. While there is no natural source with high expression found for TAPL, the B-lymphoma cell line Raji shows high endogenous expression suitable not only for cell biological but also for biochemical studies (Uebel et al., 1997; Gorbulev et al., 2001; Chen et al., 2003). Both transporters are stably and transiently expressed in mammalian cell lines (Demirel et al., 2012; Hinz et al., 2014). Moreover, good expression in *Spodoptera frugiperda* cells with the baculovirus expression system and even stably integrated in the genome in *Drosophila melanogaster* cells is reported (Meyer et al., 1994; van Endert et al., 1994; Schoenhals et al., 1999; Wolters et al., 2005). In baker's yeast only very small amounts of TAP were produced (Urlinger et al., 1997), whereas both transporters are expressed to a decent level in *Pichia pastoris*, high enough for biochemical and structural studies (Schölz et al., 2011; Parcej et al., 2013; Zollmann et al., 2015; Oldham et al., 2016a). For TAPL, a single step purification via His-tag was applied (Zhao et al., 2008), whereas the heterodimeric TAP complex is isolated in a two-step, orthogonal process using a His-tag and a streptavidin-binding-peptide tag to capture only heterodimeric complexes (Parcej et al., 2013). More recently, TAP was isolated by the viral inhibitor ICP47 ending with a thermostable, inhibited complex well-suited for single particle cryo-EM analyses of the native MHC I peptide loading complex (Herbring et al., 2016; Blees et al., 2017). Functional reconstitution is a prerequisite for mechanistic studies. A critical step is the detergent used for solubilization. Interestingly, TAPL is less dependent on the detergent since digitonin as well as n-dodecyl-β-D-maltoside (DDM) restores its activity (Zhao et al., 2008), whereas TAP is only functionally solubilized in digitonin and the steroid based glycol-diosgenin in the range of 80 different detergents tested (Herget et al., 2009; Lehnert et al., 2016). Digitonin solubilized TAP is enriched in phosphatidylethanolamine and phosphatidylinositol. Remarkably, TAP solubilized in DDM and therefore inactive could regain its activity if reconstituted in membranes rich in these phospholipids (Schölz et al., 2011).

## Author contributions

All authors listed have made a substantial, direct and intellectual contribution to the work, and approved it for publication.

### Conflict of interest statement

The authors declare that the research was conducted in the absence of any commercial or financial relationships that could be construed as a potential conflict of interest.
